# Computational models of peripersonal space representation

**DOI:** 10.1016/j.plrev.2025.07.002

**Published:** 2025-07-03

**Authors:** Tommaso Bertoni, Ishan-Singh J. Chauhan, Jean-Paul Noel, Andrea Serino

**Affiliations:** a MySpace Lab, Department of Clinical Neurosciences, University Hospital of Lausanne, University of Lausanne, Lausanne, Switzerland; b Translational Neural Engineering Laboratory, Neuro-X Institute, Ecole Polytechnique Federale de Lausanne (EPFL) 1015 Lausanne, Switzerland; c Department of Neuroscience, University of Minnesota, Minneapolis, United States

**Keywords:** Peripersonal space, Computational model, Body representation, Statisticalregularities, Space and time

## Abstract

Peripersonal space (PPS) is the region of space near the body, the multisensory interface where interactions with the environment predominantly occur. This space is represented by a specialized neural system that integrates tactile and external stimuli as a function of their distance from the body. Previous studies uncovered plastic and dynamical properties of PPS representation, links between PPS encoding and higher-level cognitive functions (e.g., social cognition), as well as its alterations in neurological and psychiatric disorders. These findings have expanded the definition of PPS and have led to the development of an array of computational models of PPS, addressing the *why* and *how* of PPS encoding. Although computational models are crucial for advancing our mechanistic and functional understanding of PPS representation, no prior work has reviewed these models. Here, we address this gap by analysing computational models of PPS, and proposing a taxonomy to classify them based on their level of description, capacity to reproduce empirical findings, and ability to generate novel predictions. This effort leads us to propose that PPS may be best understood as a system that detects spatiotemporal regularities in body-environment interactions, in order to predict potential future interactions. Hence, we suggest re-defining PPS as a unified spatiotemporal field that integrates not only spatial dimensions, but also temporal ones.

## 1. Introduction

### 1.1. What is peripersonal space?

We keep our loved ones close and push threats away. These physical interactions with the world happen within a sector of space close to our bodies, our Peripersonal Space (PPS). This notion was introduced in systems neurophysiology in the late 70 s and early 80 s [1–4]. PPS defines the spatial extent of the visual receptive field (RF) of macaques’ premotor neurons that respond both to tactile stimulation on a given body part, and to visual stimuli presented around that same body part [1,2,5–8]. In addition to premotor areas, neurons with similar properties were then observed in the posterior parietal cortex, in particular in the ventral intraparietal sulcus [5, 9], and in the putamen [10]. Later, studies in humans demonstrated a similar dedicated encoding of PPS as distinct from far space. Namely, neuropsychological patients suffering from crossmodal extinction [11,12], and studies on healthy individuals using crossmodal congruency tasks [13–15], demonstrated that tactile processing is most readily modulated by external stimuli close to the body, rather than far from it. This suggests that, in primates, near and far space have distinct neural representations.

Following these early findings, authors have defined PPS as the “reachable space” [16], as a “defensive space” [17–19], as engendering a contact prediction mechanism [20,21], or more recently, as an “action field” representing the value of potential actions [22]. In addition, others have highlighted how the near space is particularly important for social cognition [16,23], suggesting a link between PPS and interpersonal space (i.e., the distance that individuals are comfortable with keeping during social interactions [24]). Finally, it has been suggested that PPS mechanisms integrating multisensory bodily stimuli (i.e., touch) with external cues (i.e., visual or auditory) support key components of bodily self-consciousness, such as body ownership and self-location [25–29]. In sum, while the early description of PPS was firmly grounded in single cell recordings (i.e., a fronto-parietal network integrating multisensory bodily stimuli with external stimuli as a function of their distance from the body), the term PPS has since been expanded to encompass a much broader semantic and theoretical landscape. Here, we will examine PPS through the lens of computational models, trying to bridge from the early neurophysiological descriptions to the array of functions now ascribed to PPS.

### 1.2. Computational models as a bridge across observations related to PPS

In recent years, several computational models of PPS have been proposed, addressing both its neural mechanisms and functional properties. These studies, attempting to bridge from behavioural observations to neural implementations, have by and large either adopted artificial neural networks (ANNs) or normative mathematical models (NMMs) to emulate some of the key properties of PPS. We believe that adopting a modelling perspective can advance our understanding of the PPS system for a variety of reasons. First, although not exclusive to computational models, it encourages a rigorous, synthetic and explicit formulation of the system’s functional characteristics, as this is a necessary starting point for any computational model. Second, computational models tend to naturally translate these functional principles into sets of well-defined formal operations, that can be exploited to generate novel hypotheses, experiments and further model refinement. Third, some modelling approaches also provide insights into the physiological implementations of these formal operations which could hardly be inferred from limited existing electrophysiological evidence. In sum, the modelling perspective helps integrate the heterogeneous factors shaping PPS representation into a coherent mechanistic framework. This allows to refine our theoretical understanding of PPS, enables novel predictions guiding future experiments, and helps explore pathologies by simulating changes in physiological parameters.

Despite these valuable contributions and the well-established importance of computational modelling for neuroscience, a comprehensive review of the existing computational accounts of PPS remains notably absent. The present work aims at addressing this gap, and by doing so, to highlight the value that computational models can bring to the field of PPS. In the next sections, we first summarise the key properties of PPS that ought to be addressed by computational models. Then, we focus on reviewing computational works describing the main functional properties of PPS. After closely examining points of convergence and divergence between computational models, we conclude by suggesting that the concept of PPS may be reframed as a system detecting spatiotemporal regularities in ongoing body-environment interactions (as hinted in [30,31]) and using these regularities to make predictions about potential self-environment interactions [20]. In this context, we propose to redefine PPS as a unified neural representation encompassing not only the dimension of space, but also that of time.

### 1.3. Inclusion criteria and key functional properties of PPS

We begin by defining the minimal characteristics of a computational model to be considered a “PPS model”, and thus the minimal characteristics needed be included in the present review. In our view, the minimal property to define the PPS system is the distance-dependent integration of tactile and visual/auditory information, with stronger integration for stimuli near the body [11,18,32]. We thus reviewed any computational model focusing on such form of multisensory integration. Below, we briefly summarize the main additional properties of PPS representation, focusing on those which have been the subject of computational modelling. For more comprehensive and general reviews regarding the properties of PPS, see [11,18,32–34].

**Fig. 1.**
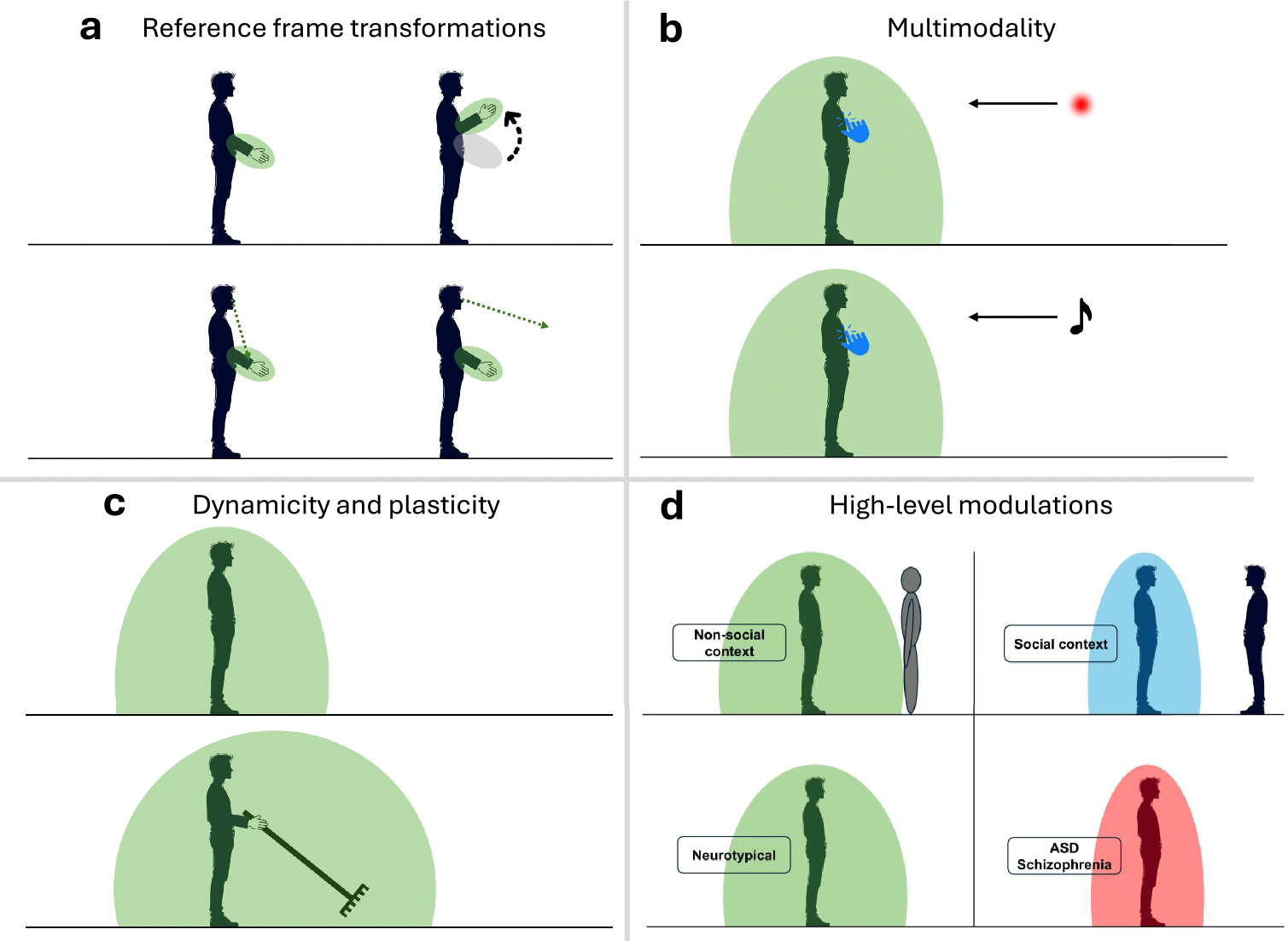
Key properties of PPS representation. (a) The RFs of PPS neurons are spatially anchored to body parts in space (irrespectively of gaze), requiring reference frame transformations across sensory modalities. (b) PPS representation is multimodal, involving the integration of tactile information with external stimuli in different modalities. PPS is thus often studied through the use of tactile stimuli alongside external stimuli, which can be visual (top) or auditory (bottom). Of note, looming stimuli elicit stronger PPS responses. (c) PPS representation changes dynamically (in response to the properties of ongoing stimuli) and plastically (in response to repeated stimulation patterns). Here, the example of PPS extension following tool-use is shown. (d) Overall, PPS representation reflects higher level cognitive factors, amongst which only those linked with psychiatric disorders have been investigated through computational models. Here, we show as examples the facts that PPS shrinks in the presence of others (top) or in psychiatric disorders (bottom).

First, tactile and visual RFs of PPS neurons in monkeys, and PPS-like responses in humans, are kept in register in body-centred reference frames. For instance, if an animal moves its arm, the visual RFs of PPS neurons responding to touch on the arm stay anchored to it and shift coherently. Notably, visual RFs are unaffected by movement of the animal’s gaze [6,35]. This also applies to behavioural proxies of PPS representation such as reaction times [30]. Thus, PPS representations are body-part centered. Since some of the sensory inputs underlying PPS representation are not encoded in body-centered coordinates, computing PPS requires reference frame transformations (Fig. 1a). These transformations can be described as the combination of two steps: a coordinate transformation, such as converting proprioceptive inputs from joint angles to Cartesian coordinates; and a coordinate alignment or origin shift, for example, from a trunk-centered to a limb-centered reference frame.

Second, external stimuli triggering PPS-like responses can also be auditory [36], body-part centered encoding implies a key role for proprioception [36], and evidence shows that PPS responses are also influenced by vestibular inputs [37–39]. Thus, PPS representation is *multimodal*, as it encodes the position of external stimuli regardless of sensory modality, and the anchoring of RFs depends on a host of senses, including proprioceptive and vestibular inputs (Fig. 1b).

Third, PPS representation changes as a function of current and past interactions with the environment (Fig. 1c). We will refer to changes occurring in response to ongoing stimuli as *dynamic*, and changes following repeated exposure to stimulation patterns as *plastic* [34]. PPS varies dynamically as a function of “instantaneous” properties of the external stimulus, such as its velocity [6,40], its valence [41,42], or its direction (looming vs. receding; [43]). In addition, the extent of PPS, i.e. the distance at which a visual stimulus significantly affects tactile processing, depends on the distance of the visual stimulus in immediately preceding trials [44]. On the plastic side, using a tool extends PPS-like responses [45] on the timescale of minutes, or even permanently for long-term users [46,47]. Similarly, upper limb immobilisation [48] or sensorimotor impairments [49] contract PPS around the affected limb.

Finally, PPS properties are affected by various higher-level aspects, such as social cognition and neuropsychiatric and neurodevelopmental conditions (Fig. 1d). Computational models have mainly focused on the latter. For instance, young adults on the autism spectrum show a smaller and sharper PPS than that of neurotypical individuals [50,51]. Additionally, their PPS does not adapt to social context, contrary to what was observed in neurotypical individuals [52]. In schizophrenic patients, PPS is also generally described as smaller than in neurotypical populations [53], but see also [51] for a report of null effects.

## 2. Computational models of peripersonal space

The key reviewed properties of PPS representation vary widely, spanning from basic neurophysiology to abstract cognitive functions. In an attempt to characterize the models’ epistemological value across such a broad spectrum, we organized the existent computational models of PPS along three axes. The first axis refers to the level of description that the model is focused on, in a categorization based on Marr’s theory of perception [54,55]. The second axis reflects the known properties of PPS representation that each model can explain. The third axis refers to the ability of the models to generate novel, testable predictions.

### 2.1. Description levels of PPS computational models

In this section, we will briefly present each reviewed work in terms of Marr’s levels of description they focus on [54,55]. Marr proposed that neural information processing can be analysed at three distinct levels. At the most abstract level, the system can be described in terms of its “purpose”, that is, which evolutionary advantageous task it helps the organism achieve. This description level was originally termed “computational level”, but here we will refer to it as the “functional level”, to avoid confusion with the general focus of the present review on computational models. The second description level, the “algorithmic level”, refers to the formal operations that are carried out by the system to achieve its goal, and the neural representations it uses to this aim. It is important to note that, while all computational models by definition consist of a set of formal operations, their epistemological value on the algorithmic level depends on whether these operations provide a compact and interpretable description of behaviour or neural activity. The third description level, the “implementation level”, refers to the physical substrates that the system uses to carry out such computations, that is, the underlying neurophysiological structures and mechanisms.

**Fig. 2.**
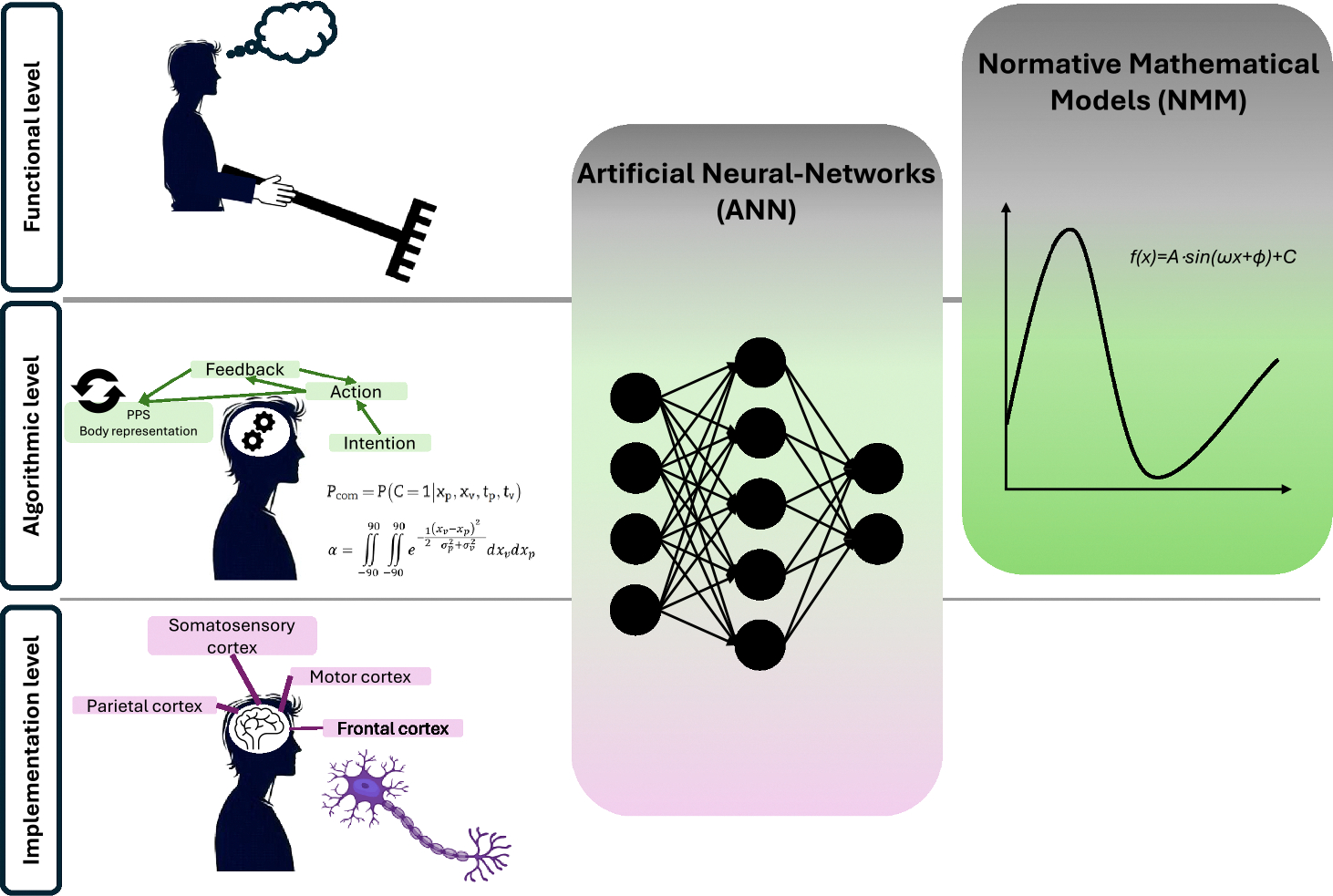
Marr’s levels of analysis and computational model classes. David Marr’s functional level consists in specifying the goal and utility of the neural process in question (for instance, in our case, PPS could be considered as the system allowing interactions with the environment through the body or artificial effectors such as a tool). The algorithmic level describes the formal operations that are performed to achieve such goal, and can often be cast in a mathematical framework (for instance in our case, a chain of computations spanning over motor intention, sensory perception and action, leads to higher order body and PPS representations). The implementation level describes the underlying neurobiological processes and structures (for instance in our case, premotor and parietal cortices as well as the putamen are key brain areas underlying the PPS system). ANNs directly model the behaviour of simulated neurons, thus providing insights about the implementation level. At the same time, their overall behaviour can sometimes be translated in more abstract mathematical formulas, or can be to some extent tuned to achieve a given goal, providing some information about the algorithmic and functional levels as well, though to a lesser extent than NMMs (as indicated by the colour intensity). NMMs are instead directly conceived by starting from a goal that the system should achieve, and they translate this goal into a set of formal mathematical operations, being usually fully agnostic on the neural substrate implementing them. Thus, they provide information about the functional and algorithmic levels.

While Marr’s levels offer a useful analytical separation, they are not independent in reality. The functional level sets the goals of the system and thus constrains the range of viable algorithmic and implementation strategies. Conversely, to be epistemologically meaningful, what is functionally plausible must also be constrained by what is biologically and algorithmically feasible. Similarly, the algorithmic level must not only operationalize the functional goal but do so in a way that can, at least in principle, be realized by neural structures. Thus, the three levels should ideally inform and constrain each other. The most robust models are those that maintain coherence across levels, strengthening their interpretability and explanatory value and offering more compelling support for their validity. Thus, while ideally models should bridge all three levels of description, this is often not feasible due to current limitations in biological knowledge. Nonetheless, valuable insights can still emerge from models that address mainly one or two levels. Overall, Marr’s levels provide useful guidance in evaluating the field of applicability of each model (see Fig. 2 for a schema), a crucial aspect for correctly interpreting results from computational works.

Most works described here use ANNs to model PPS representation, attempting to make PPS properties emerge from the behaviour of simulated neurons. While all ANNs formally aim to make the functional and algorithmic levels directly emerge from the implementation level, there is broad variability in how closely they mimic actual neurophysiology, hence impacting the applicability of their predictions. ANNs also highlight a key distinction between the algorithmic-level and the implementation-level. The implementation-level mathematical operations carried out by an ANN are distributed across the network’s weights and activations and have no straightforward, compact interpretation. Thus, they provide epistemological value at the algorithmic level only if their properties can be traced back to an interpretable mathematical principle.

Another, less represented, category of models treated here are NMMs (e.g., Bayes optimal models, models that are built to be statistically optimal and then used as benchmark for querying actual human behaviour). Unlike ANNs, NMMs do not directly describe the underlying neural computations, but mathematically formalize the optimal behaviour that an agent should follow to achieve a goal under a certain set of assumptions (i.e., the most evolutionarily advantageous). Thus, their inherent focus is on the functional and algorithmic level, and they cannot usually provide implementation-related predictions. However, they can model the functional and algorithmic levels based on a rigorous application of evolutionary principles, typically using fewer free parameters than ANNs.

**Fig. 3.**
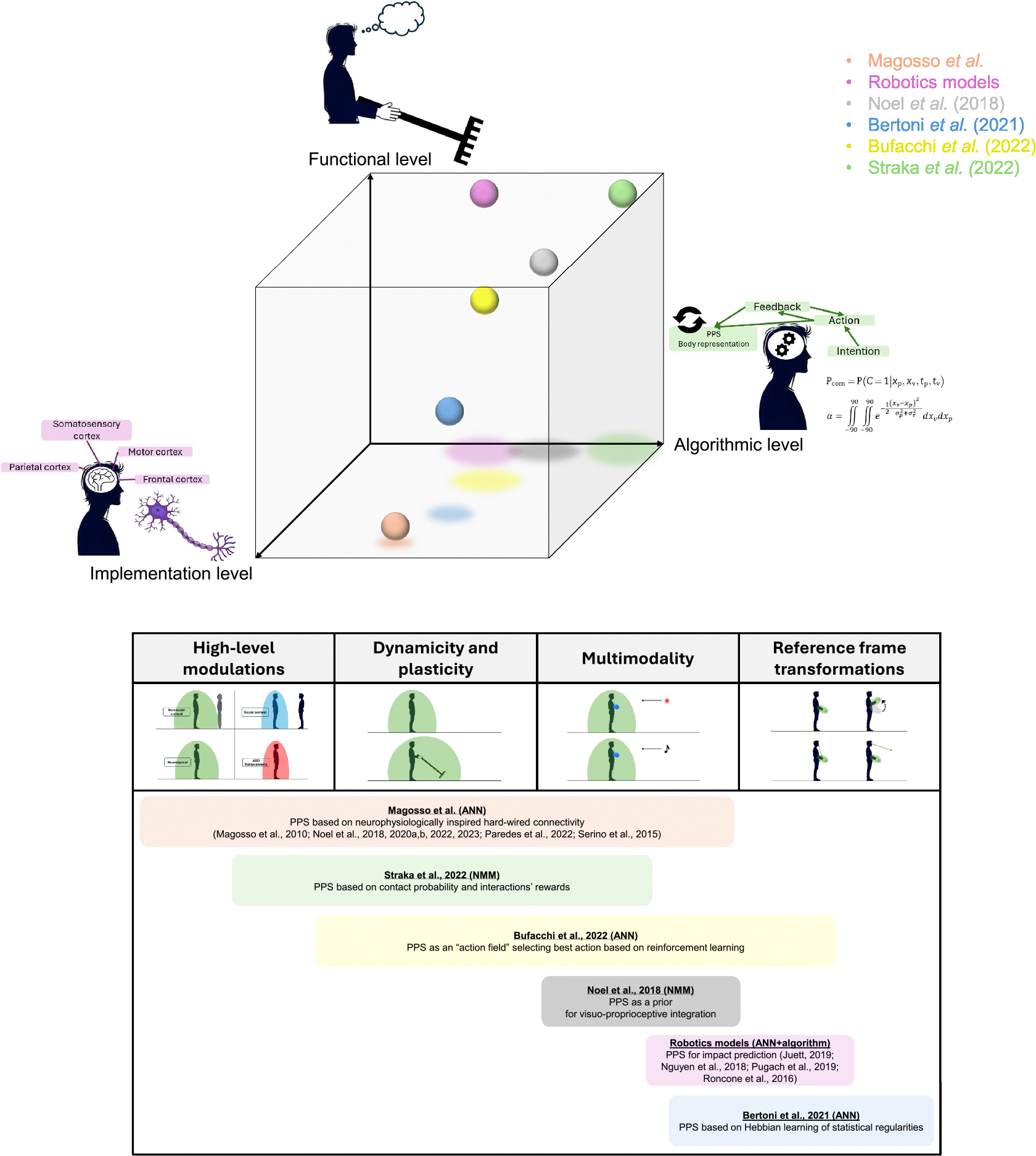
Summary of reviewed models’ main description levels, characteristics and predictions. Top: models represented in a 3D space, according to how much they focus on each of Marr’s description levels. Models from the robotic field [66–69] have been grouped together. Models which are a variation of the original work from Magosso et al [40,44,60–63,74] have been grouped together. Shades indicate the projection of each model on the horizontal plane, and their size is proportional to their height on the functional axis. Bottom: models positioned according to which key PPS properties they address.

In presenting the reviewed models, we will loosely order them from the most implementation-oriented to the most functional-oriented, without claiming strict objectivity in this classification (see Fig. 3 for a graphical summary of the reviewed models). In the models most focused on implementation, populations of artificial neurons are organised to mimic the processing of populations of neurons observed *in vivo*, according to the type of information they encode and to the way they are interconnected.

Computational works in the early 2000s first attempted to model PPS properties – i.e., distance-dependent integration of tactile and visual/auditory information based on the overlap of multisensory (visuo-tactile) receptive fields into a common reference frame [56–58]. They characterized VIP neurons showing fully body-centered responses to tactile stimuli and visual responses that were eye-centered (sensitive to eye position), head-centered (not affected by eye position) or mixed. By applying recurrent basis function network, they were able to model the emergence of overlapping, body centered visuo-tactile responses (^57^; see [59] for a similar finding, yet with a different computational approach, for visuo-tactile hand responses). This approach was seminal to investigate PPS-related computations. However, it was not exploited to simulate the other PPS properties described above, and thus these works cannot be evaluated within the framework proposed in this review.

Magosso’s model [60] (and its close derivations [40,44,61–63]) featured populations of unisensory neurons encoding tactile inputs on a part of the body (e.g., the hand) and populations of visual or auditory unisensory neurons encoding the position of an external stimulus. These unisensory layers were connected with a multisensory population of neurons integrating both unisensory inputs. To reproduce the basic spatial property of PPS representation, i.e., stronger integration for stimuli near the body, the synapses from the unisensory to the multisensory neurons were designed such that unisensory neurons with RFs on or close to the body were connected with strong synapses to multisensory neurons. Conversely, unisensory neurons with RFs far from the body were connected with weak synapses to multisensory neurons. In this way, PPS representation, i.e. distance-dependent multisensory integration, emerges from the model architecture, thus reproducing both behavioural findings and basic observations about the neurophysiology of PPS neurons. In this class of models, the functional level of description purely emerges from network architecture, and does not explicitly act as a driving force in building the model.

Importantly, Magosso’s models encoded external stimuli in pre-computed body-centered coordinates, thus the spatial “anchoring” of RFs to body parts is an assumption, rather than a feature of the model’s approach [60]. To explicitly model reference frame transformations from external to body-centered coordinates, Bertoni et al [30] encoded information about external stimuli in external or retinal coordinates (as originally computed by unisensory systems), and added an additional population of unisensory neurons coding (through proprioception or vision) for the position of the body part where tactile RFs are anchored (e.g., the hand). Most importantly, in Bertoni’s model [30], the synaptic connectivity between unisensory and multisensory neurons was not hard-wired as in Magosso’s class of models, where synaptic weights were designed to obtain the desired properties. Instead, synaptic connectivity started from random weights, and was tuned based on Hebbian plasticity (where synaptic changes are based on the co-activation of connected neurons) during ecological interactions within a simulated environment. The model’s plasticity rule was designed to learn the statistical regularities across sensory inputs, based on the functional level assumption that neural systems act as statistical models of the environment.

Bufacchi and Iannetti conceptualised PPS as an action or value field [22]. Accordingly, their neural network model [64] focused on how PPS representation emerges from computing value in body-environment interactions, by implicitly predicting the expected (discounted) reward of future events linked to a given starting configuration. Rather than focusing on the perceptual processing of external stimuli to shape its synaptic weights, this model simulated an artificial agent in its environment, and the potential actions that the agent may perform to interact with external stimuli. In each simulation instance, the model learned to select between a set of predetermined actions for the one yielding the highest expected reward. Instead of Hebbian plasticity, the authors chose reinforcement learning as the key driving force to update the model’s synapses, based on whether the selected action led to a positive or negative reward. In this way, the more rewarding behaviours are favoured. This approach offers a powerful tool to reproduce the emergence of PPS, as it is driven by the functional assumption of reward maximisation, which has a clear evolutionary motivation.

A separate category of models, focusing on the functional and algorithmic level, are NMMs. Within this context, Straka and colleagues proposed a model in which the optimised behaviour is an agent’s ability to correctly avoid threats, without renouncing to approach stimuli with positive valence [65]. The balance between the positive and negative rewards of correctly or incorrectly predicting interactions’ valence was sufficient to reproduce key behavioural properties of PPS representation. Finally, Noel and colleagues [40] proposed a NMM suggesting that PPS would essentially consist in a *prior* for visuo-proprioceptive integration. In their model, visual and proprioceptive signals would be more likely to be perceived as originating from the same physical cause, and their spatial position integrated in a single estimate, if they are located within PPS.

The models so far described constitute neuroscientific tools to better understand how the brain represents PPS, and provide predictions about behaviour and neurophysiology. It is worth mentioning, however, that other computational works applied concepts of PPS representation to robotics [66–69]. These works took inspiration from neurophysiological and behavioural knowledge to guide robot-environment interactions, focusing on learning to predict impacts and generating motor commands, rather than reproducing behavioural or neurophysiological findings. In some respects, given the interdependence of Marr’s levels, this limits their explanatory value. Nevertheless, they represent an interesting opportunity to test body-environment interactions “in-vivo”, i.e., via a humanoid robot interacting with the physical world, rather than in-silico, in a simplified simulated environment. These works thus demonstrate that not only can neuroscience inform robotics, but that the opposite is possible too.

Pugach and colleagues [68] developed an ANN with plausible visual, tactile and proprioceptive inputs, although its learning schema and dynamics are engineered to optimise robotics performance rather than biological realism. The network learned the association between the “proprioceptive” robotic arm configuration and the visual location of tactile stimulation. The contributions by Roncone et al. and Nguyen et al [67,69], implemented in the same humanoid robot, were based on “RFs” centred at various “skin” locations, which learn the probability of contact of external objects based on their distance and the estimated time to contact (resulting from velocity and distance). The notion of time to contact shows how spatial and temporal features are inherently linked in a system that needs to evaluate potential interactions with the environment. These computations were not instantiated in an ANN, but in a pre-compiled algorithm directly implementing a set of mathematical equations. In Juett’s work [66], a robot learned the association between joint angles and visual information about arm position, and potential sequences of actions leading from one configuration to another, by randomly “babbling” to explore different configurations. Again, such learning was not implemented in an ANN, but in a pre-compiled algorithm. Currently, we have no notion of whether and how those equations would be encoded in neurons, nor on how they would emerge or develop. Thus, from an empirical and “applications” perspective, it is impressive what these artificial agents can learn from just simple interactions, and that they can provide useful insights regarding how PPS develops. However, from an epistemological perspective, especially at the algorithmic and implementation levels, these approaches may not add significant knowledge to our understanding of neural mechanisms underlying PPS.

### 2.2. Explanatory value of PPS computational models

The second axis of review is the ability of models to reproduce known properties of PPS representation. It is important to note that, especially when dealing with the complexity of ANNs, it is crucial to clearly identify the set of conditions allowing such model predictions to be generated. Indeed, determining whether these consist in a broad set of arbitrary parameters, or in a small set of justifiable and straightforward general principles, is a crucial step in assessing the epistemological value of a model. It is also important to consider at what level these predictions are valid, i.e., whether they forecast the direction of a behavioural effect, or whether they also predict a specific pattern of neural activity or functional deficit, once the system is damaged. Another important caveat with neural-network modelling is that a broad range of strategies can be used to map model predictions onto behaviour. This requires assumptions and approximations which should be considered when evaluating model predictions (e.g., see below an overview of the different strategies to model reaction time [RT] data).

As per our inclusion criteria, all the reviewed models are able to reproduce the basic feature of PPS; namely space-dependent, body-centred interactions between tactile and exteroceptive (e.g., vision and audition) sensory modalities. Behaviourally, these interactions have mainly been measured through multisensory tasks, assessing the modulation of tactile reaction times by external stimuli (e.g., [43]) or the modulation of tactile perceptual accuracy by the trajectory of external stimuli [20,29]. In terms of RT, the strength of such modulation depends on the stimulus’ distance from the body, with tactile reaction times becoming increasingly fast as stimuli get closer to the body [43]. This effect was reproduced by several of the reviewed neural-network models, using different strategies to map the activity of artificial neurons to human psychophysical measures. In Magosso’s models [60], the RT proxy was the actual time (in simulation steps) taken by the network’s tactile neuron to reach a certain activity threshold when receiving tactile inputs. External visual or auditory stimuli modulated tactile neurons’ activity through a feedforward/feedback loop to the multisensory layer. In Bufacchi’s model [64], this effect was instead approximated by measuring the network’s probability of detecting an external stimulus (as empirically shown in [20]), under the assumption that a higher detection probability should correlate with shorter reaction times. In Straka’s model [65], a similar principle was applied, and the probability of future contact computed by the probabilistic model was taken as the RT proxy.

More subtle properties of multisensory interactions related to PPS representation were also reproduced by some models, the most important being the anchoring of visual/auditory RFs to the associated tactile RF as body parts move in space (Fig. 1a). Since external (auditory or visual) stimuli are encoded in different reference frames than body-part position information, this requires performing reference frame transformations. While models such as Magosso’s [60] directly used pre-computed body part-centered coordinates as inputs, other models [30,64] explicitly accounted for reference frame transformations, by providing visual inputs in external or retinal coordinates, and limb position information through proprioceptive inputs. In Bertoni’s model, body part-centered responses were achieved both when proprioceptive inputs were encoded in Cartesian and joint-angle coordinates, indicating that the network is implicitly performing coordinate transformation and alignment [30]. However, this process is not implemented through explicit intermediate representations of transformed coordinates, but emerges from implicitly learning statistical associations across sensory modalities, showing how algorithmic descriptions can differ from their neural implementation in an ANN. In Bufacchi’s model [64], the same process emerged from learning to predict the potential reward obtained by tactile stimuli based on their visual position, coupled with proprioceptive information about hand position. In Bufacchi’s model, to reproduce hand-centered encoding of visual inputs, proprioceptive input was modelled as a simplified one-dimensional Cartesian coordinate, since reference frame transformations are not the main focus of the model. However, Bufacchi’s model may arguably generalize to more complex coordinate encodings, if appropriately trained. On a related note, Straka’s model [65] also reproduced the finding that the spatial extent of PPS varies for different body parts (see [70] for empirical data), which emerged from the fact that larger body parts have a higher probability of coming in contact with external objects.

Multimodality, another key property of PPS representation (Fig. 1b), is implicitly included in all the reviewed models, which all process “amodal” abstract information about limb and stimuli position, and have no a priori constraint in the modality in which the input stimuli are presented. This also implies that all models use an encoding schema which is too abstract to capture the known differences in neural representation across sensory modalities.

Another important aspect of PPS representation which was covered by modelling works are its dynamic and plastic properties (Fig. 1c). By adding Hebbian plasticity to the Magosso model [60], Noel et al [44] were able to explain PPS changes at the seconds-scale. The authors observed that during the classic multisensory interaction PPS task, PPS tends to expand if touch in the previous trial was associated with a far stimulus, and vice versa. In-silico experiments simulating the task performed in human participants replicated this effect. Noel et al [40] modified the original Magosso model [60] to reproduce the dynamic effect of PPS extension with increasing stimulus velocity [6]. The authors introduced a term accounting for neural adaptation (i.e., decreased excitability following prolonged activity) within the unisensory layers processing visual or auditory exteroceptive signals. This way, because of lateral inhibition between contiguous unisensory neurons, slower stimuli send weaker excitatory inputs to the multisensory neurons than faster stimuli, thus evoking a multisensory response only at closer distances. In Bufacchi’s model [64], the same effect was obtained through the spontaneous tuning of RFs to stimuli with different velocities, due to the velocity dependent timing and reliability of contact related reward. In Straka’s [65] NMM, this effect emerged from the greater uncertainty in the time and probability of contact with an object moving faster. As the model aims at minimising false predictions about potential contact, it accounts for such greater uncertainty by assigning non-zero contact probability at farther distances for fast moving objects. In summary, velocity tuning of PPS spatial properties can emerge from broadly different architectures and conceptual frameworks. While this convergence does not constitute direct empirical evidence, it suggests that the entanglement of spatial and temporal features of the processed stimuli, inherently captured by their velocity, may be a useful organizing principle for understanding PPS representation. The exact neural mechanism underlying this processing is still unknown.

To explain tool-use induced plasticity, Magosso et al [63,71] added Hebbian learning to their model and simulated tool-use “training” by simultaneously activating far-space sensitive visual (or auditory) neurons and tactile neurons. Because tactile neurons also strongly activate the multisensory neurons, this co-activation led to Hebbian potentiation of the normally weak synapses between unisensory neurons with far RFs and multisensory neurons. As a consequence, after simulated “tool-use”, stimuli presented in the stimulated region of space where the “tool” was used were able to induce a stronger multisensory effect than at baseline. Notice that since the strategy used to simulate tool use was the mere co-activation of far space coding neurons and tactile neurons, no actual tool was present. Interestingly, the effectiveness of such minimal simulation of tool use suggested that the presence of an actual tool may not be necessary to induce plasticity, leading to two novel hypotheses that were later verified empirically. First, once a plastic extension of PPS is induced via actual tool-use, stronger multisensory interaction at farther distances should be observed, independently of the presence of the tool. This was confirmed in a case-study by Magosso et al [71] who showed, after tool-use, the same level of cross-modal extinction induced by far visual stimuli irrespectively of whether the patient was holding the tool or not. The second, even stronger, hypothesis is that in order to extend PPS, it is not mandatory to use a tool, but it would be sufficient to provide synchronous tactile stimulation on the hand and visual or auditory stimulation from the far space. This hypothesis was confirmed in a behavioural experiment on healthy participants [63]. This latter report also demonstrated that in-silico synchronous auditory-tactile stimulations, in-vivo tool-use training, and in-vivo synchronous auditory-tactile stimulations, all induced a strikingly similar extension of PPS, as demonstrated by the same speeding effect of tactile RT induced by far auditory stimuli. Tool-use induced plasticity could also be replicated in the reinforcement learning approach to PPS synaptic plasticity by Bufacchi et al [64]. Here, the authors simulated tool-use and rewarded the agent’s actions not only when object location coincided with limb location, but also when it coincided with the location of the tip of the tool.

Some of the proposed computational models of PPS are also able to account for differences in PPS encoding due to higher-level factors (Fig. 1d), the most prominent being neuropsychiatric and neurodevelopmental conditions. Work inspired by the Magosso model has explicitly accounted for aberrant PPS encoding in autism [62] and schizophrenia [61], and has mostly suggested that these alterations are embedded within PPS networks themselves. An important innovation in Noel and colleagues’ work [62] with respect to prior implementations of the Magosso model [60] is the implementation of true model fittings. The authors were able to determine which model parameters best accounted for empirically measured PPS modulations across conditions and individuals. They argued that remapping during social contexts [52] was better explained by a modulation of the input-output function of multisensory PPS neurons, rather than by modulation of feedback connectivity from multisensory to unisensory neurons. That is, it is not necessary to postulate an effect due to an auxiliary network computing value (as in [64,65]) and putatively impacting the activation function of all neurons (unisensory and multisensory) and/or synaptic connections (feedforward and feedback). Next, to account for the smaller size of PPS in autism relative to neurotypical controls, the authors hypothesised an anomaly in the excitatory (E) to inhibitory (I) ratio of lateral connectivity within unisensory areas. This E/I imbalance is a well-established hypothesis in the autism literature [72], and thus the hypothesis in Noel et al [62] would place an anomaly of PPS size in autism within the broader framework of the condition. The E/I imbalance was indeed able to recapitulate idiosyncrasies in PPS size in individuals with autism. More importantly, when the authors then studied social contexts, modulating the input-output function of multisensory PPS neurons, within the autism-like E/I regime, they noted that modulations in the input-output function were ineffective in generating a change in PPS size - just as behavioural data had shown. Paredes and colleagues [61] similarly showed that E/I imbalances can account for smaller PPS sizes in schizophrenia, and further suggested that a decrease in the synaptic density of feedforward and feedback connections (i.e., synaptic pruning) can account for sharper PPS boundaries in schizophrenia [53].

Although not explicitly mentioned in our schema (Fig. 1d), a few other results about the interaction between PPS and higher-level cognition are worth mentioning. A first high-level PPS modulation reproduced by certain computational models is stimulus valence dependency. Bufacchi’s model [64] reproduced the effect that stimuli with more valence elicit stronger responses from PPS neurons [73], consistently with the authors’ view that PPS representation is an “action field” for selecting the most rewarding actions. Interestingly, the model also predicted that, in order to encode appropriate actions depending on stimulus valence, distinct subnetworks serving different classes of actions would develop. This closely resembles neurophysiological findings in non-human primates, which identified areas more linked to defensive (such as VIP and F4) or reaching/grasping movements (MIP, AIP, 7B, F5). Another work which touches on the theme of stimulus valence, and the related notion of priming defensive or appetitive reactions, is the paper by Straka and colleagues [65]. Their model predicted that the size of PPS would depend on the penalty for a false negative contact prediction, becoming larger as such penalty increased. This is reminiscent of previous empirical findings [41]. Also worth mentioning is the link between PPS representation and key components of bodily self-consciousness, namely body ownership. In Bertoni’s work [30], the same ANN trained to reproduce key features of PPS representation could reproduce a known behavioural correlate of body ownership in the rubber hand illusion. In humans, tactile stimuli on the hand coupled with visual stimuli on a fake hand at a spatial location shifted from the hand induce a shift of the perceived location of one’s own hand, and a subjective feeling of ownership for the fake hand. Simulating such stimulation pattern in the ANN, the network’s proprioceptive encoding also shifted toward the location of the “fake hand.”

### 2.3. Generating new predictions

A last, but not least important, axis along which a model can be described is its ability to generate novel predictions, rather than simply reproducing existing data. This feature is crucial in the context of neural network models (i.e., the majority of models within the PPS field), where the number of parameters that can be arbitrarily tuned to reproduce a given known behaviour is extremely large. In such case, the ability to generate new, testable predictions that are confirmed through empirical testing provides much more compelling validation than merely reproducing known results.

The first, easily testable category of predictions are behavioural ones. We mentioned two examples of predictions generated by Magosso’s model to explain PPS tool-use plasticity via Hebbian learning, that have been empirically demonstrated (^63,71^; see Section 2.2). Another example of novel, yet this time untested, behavioural prediction is found in Straka’s NMM. This account predicts that noisier stimuli should increase the extent of PPS, to reflect the greater uncertainty of potential contacts. The authors also suggest that the relation between stimulus uncertainty and PPS size should be stronger for smaller body parts, for which the influence of uncertainty about the stimulus on the probability of contact is greater.

A second category of predictions concerns neurophysiological properties of PPS representation. For example, behavioural predictions about tool-use plasticity could be expanded on the neurophysiological side, by comparing training-induced changes in resting-state or during-task functional connectivity (assessed via EEG or fMRI) with model predictions about plastic changes in synaptic strength. Looking purely at the neurophysiological side, a rich source of untested predictions lies in models which plastically learn their synaptic connectivity. Compared to NMMs or hand-wired ANNs, these models implicitly provide an additional class of predictions: the emergent synaptic organisation and the characteristics of RFs. These predictions hold great potential in addressing a key limitation of artificial neural networks (ANNs): the large number of free parameters, which allows for behaviour reproduction without necessarily mimicking the actual neurophysiological mechanisms (overfitting). Directly comparing the receptive fields (RFs) learned by these models with those observed *in vivo* offers a more robust and compelling validation approach, since the higher level of granularity of electrophysiological data makes overfitting less likely. These predictions, though extremely valuable, remain largely untested. This would imply carefully mapping the response pattern of PPS neurons in a broad range of conditions, and testing whether neurons with matching response patterns can be found in an ANN model. Although seminal studies in non-human primates have provided some evidence about the basic properties of PPS neurons’ RFs, the complexity of ANNs allows for a broad margin in generating novel untested predictions. Indeed, most neurophysiological research on the PPS system was conducted in pioneering years where high-throughput, systematic mapping of RFs was not routinely performed.

One example of neurophysiological predictions stemming from Bufacchi’s work [64] is the previously discussed segregation of the PPS network in subsystems, based on the encoded action class. This has been previously observed in neurophysiological studies, but Bufacchi’s model additionally predicts that such functional organisation may emerge naturally from neural architecture and the necessity to efficiently encode information. Specifically, the more the layers of the PPS network reduce in size as information travels upstream in the neural hierarchy (thus “compressing” incoming information), the more functional segregation in sub-networks is observed in the model, and should likewise be observed in-vivo. Bufacchi’s model [64] approach to velocity modulations also provides the interesting untested prediction that the spatial extent of PPS neurons’ RFs should correlate with their velocity tuning, with larger RFs in neurons tuned to faster stimuli (yet another hint at the entanglement between spatial and temporal features in PPS representation). On a related note, Noel’s modification of Magosso’s model addressed stimulus velocity by assuming that neural adaptation could underlie velocity tuning of the PPS system [40]. This suggests that PPS neurons’ excitability should decrease with the time of exposure to external stimuli, a prediction that could in principle be tested in neurophysiological experiments. Bertoni’s model predicted that multisensory PPS neurons would spontaneously segregate in populations which are excited or inhibited by tactile stimuli. The first would exhibit overlapping proprioceptive and visual RFs, and the latter would exhibit anti-overlapping RFs. Thus, to provide strong support for the mechanism proposed by Bertoni et al., one could demonstrate the co-existence of such neurons within PPS regions. These predictions resonate with evidence from different yet related domains such as multisensory neurons in the superior colliculus showing supra- and sub-additive responses [75,76], or parietal neurons encoding visual information in eye-centered, head-centered or intermediate reference frames [57,58].

A third category of untested predictions concerns pathological changes in PPS behavioural or neurophysiological correlates. An interesting and testable prediction can be generated according to the E/I imbalance and synaptic pruning hypotheses formulated to explain PPS alterations in autism [51,62] or schizophrenia [61]. In healthy individuals, it has been shown that PPS remaps quickly, such as within trials (i.e., based on the velocity of incoming stimuli), [40] or across trials [44]. In other multisensory domains (e.g., the perception of synchrony across multimodal stimuli), it is known that individuals within the autism spectrum show a reduced trial-to-trial recalibration of multisensory percepts [77]. Thus, future work may test whether individuals within the autism spectrum also show an alteration of the rapid recalibration of PPS (across trials or within a single trial), as they do within other sensory modalities, and whether models of PPS in psychopathology (i.e., E/I balance, synaptic pruning) would account for these effects.

### 2.4. How to determine a guiding principle?

Objectively summarizing PPS computational models within a unified framework poses a challenge, as the optimal approach to modelling PPS representation varies depending on the specific focus and objectives of the study.

Focusing on the implementation level, the concept of biological realism becomes central. Biological realism entails at least three fundamental aspects: architectural fidelity, neural dynamics realism, and the biological plausibility of the learning mechanism. Architectural fidelity pertains to how closely the functional connectivity of modelled brain regions resembles that of actual brains. Neural dynamics encompass how detailed and close to biological evidence are the rules of neural excitation/inhibition and their temporal evolution. All ANN models reviewed here used rather simple activation functions (e.g., none of them is a spiking neural network). However, deep learning inspired models (e.g., [78]) showed a surprising ability to reproduce empirically observed RFs with deeply simplified activation functions, suggesting that accurately modelling single neuron dynamics may not be necessary to obtain accurate predictions. Finally, the choice of learning mechanisms, e.g., Hebbian plasticity vs. reinforcement learning, requires careful consideration. As mentioned in the introduction, Marr’s levels are far from independent and should inform and constrain each other. Decades of work on PPS representation have provided important insights into its functional (e.g., contact prediction [21,65], action fields [22]) and algorithmic aspects (e.g., Bayesian inference [30,65], reinforcement learning [64]), while linking PPS to more general principles in neuroscience. Implementation focused models should strive to integrate, and possibly reconcile, these valuable yet partially diverging perspectives.

Moving to the algorithmic level, the focus shifts toward its most meaningful output: behavioural predictions. Since different algorithmic implementations of the same functional objective can lead to very similar behavioural predictions, careful assessment is crucial to select the best model. In particular, it is fundamental to consider not only whether such predictions broadly align with empirical results, but also whether they can reproduce subtle properties of behaviour with great detail. For example, accurately predicting the full probability distribution of individual trials rather than just average values offers a more compelling account. Since the epistemological value of models focusing on the algorithmic level is mainly to provide subtle behavioural predictions, a key aspect to consider is whether a given model significantly outperforms alternative models. This requires non-trivial assessments and comparisons of model predictions. As evident in NMMs, predictions at the algorithmic level often stem from assumptions at the functional level, supporting the importance of integrating and reconciling different functional interpretations of PPS representation. At the same time, algorithmic considerations maintain a bidirectional relationship with the implementation level. In some cases, algorithmic insights can be extracted by reverse-engineering implementation-focused models such as ANNs. Conversely, ANNs that are explicitly designed to implement empirically supported algorithmic principles in a biologically realistic way offer a strong epistemological advantage.

Besides considerations on the implementation and algorithmic level, even more critically are perhaps those pertaining to the functional level, as they tightly constrain both other levels of analysis. These can be arguably summarised to one central question: what precisely are we seeking to model when we say we are modelling PPS? Or more specifically: what are the functional assumptions underlying the reviewed models? Can a unified framework be derived from these assumptions? In our view, any interpretative effort should start from defining how the PPS system specialises to contribute to the overarching purpose of the nervous system: safeguarding survival in a dynamic and ever-changing environment. A key feature enabling the brain to achieve this goal is its remarkable ability to efficiently process information across varying spatial and temporal scales [79]. The brain greatly “compresses” information for far spatial and temporal scales, and has a more detailed representation of the near space and present time. Thus, it is reasonable to assume that different systems process information at different spatial and temporal scales. In this view, we believe that a useful delineation of the PPS system is to specifically refer to the system processing body-related information at the small spatiotemporal scale of body-environment interactions, “here and now”.

## 3. Spatiotemporal statistical regularities as a possible overarching framework

Indeed, independently of their architecture or their computational framework, the common factor underlying all the above-described models of PPS is arguably the brain’s necessity to predict interactions between the body and the environment, in order to prime appropriate reactions. In this context, we believe that natural statistics of such interactions play a crucial role in the emergent properties of PPS representation. Such probabilistic factors can be explicitly modelled, such as in normative, intrinsically probabilistic, models (e.g., [65]). They can also emerge implicitly from the more complex dynamics of ANNs via multisensory integration processes (e.g., [30]) or action-perception loops governed by reinforcement learning [64]. As these body-environment interactions are primarily conceptualised in terms of proximity between external stimuli and the body, their first key aspect concerns spatial features. Accordingly, PPS and its neural models are mainly conceptualised in terms of spatial layouts - e.g., the location or extent of neurons’ RFs. Yet, these interactions are inherently entangled with temporal features. Indeed, in a probabilistic framework, the potential for interaction is gauged not only by the physical distance of the stimulus, but also by the timing of appearance of this stimulus at a given distance. Despite the fact that it seems obvious that the spatial and temporal dimensions are closely intertwined, there is no or very minimal notion of temporal RFs (i.e., RFs tuned to events at a given delay from the present) in the neural instantiation of PPS [80]. Indeed, spatial properties are more intuitive to reason with, and have been the focus of most behavioural and neurophysiological investigations. However, in our perspective, these features should be seen as the tip of the iceberg of a more complex representation where spatial and temporal features are entangled.

To make sense of the relationship between spatial and temporal components of the PPS system, it may be useful to increasingly reason in terms of body-environment relationships. These relationships are captured by multisensory interactions between touch - the modality that defines and senses the physical boundary between our body and the outside - and the distal senses, primarily vision and audition, which dynamically process information related to external stimuli. In this framework, proprioceptive and vestibular inputs would play a crucial role in aligning the reference frames associated with these different modalities. However, if it only responded to spatial regularities without a temporally predictive component, the PPS system would obviously only respond when the external stimulus, as coded by visual or auditory cues, touches the body, that is, when the unisensory visual/auditory and tactile RFs fully overlap. Thus, the mere observation that ‘classical’ PPS RFs extend beyond the body suggests that a temporal predictive component of future contact may be embedded in the PPS system. In a broader sense, a key characteristic of brain function is its ability to build predictive models of the environment through learning and experience, by building associations between multiple inputs based on their statistical regularities. Applying this framework to the PPS system, i.e., reasoning in terms of potential for interaction rather than actual contingencies, would mean representing body-environment relationships in terms of interaction probabilities. Indeed, in a space-time perspective, there is no distinction between contingencies between co-occurring events, and contingencies between events which occur at different times.

An organism which is able to predict contact with the body in space and time has massively higher chances to survive, as it is able to prepare a response before being touched by an external stimulus, whether it is a potential threat to avoid or an interesting target to reach. This short-term predictive ability may further generalize to a longer temporal scale, becoming the basis for long-term planning. For these reasons, it is likely that such a neural system has emerged early in evolution and should be greatly preserved in current species. Thus, although PPS neurons have been described in such terms up to now only in primates, it is reasonable to expect a similar mechanism in a wide array of living organisms. From a phylogenetic perspective, it would be extremely interesting to understand whether evolution has selected a unique mechanism to perform this function, or whether multiple solutions have arisen across taxa. On the other hand, studying how such a mechanism emerges at the individual level through development, as a function of interactions with the environment, is key to fully understand its neural implementation and its impact on whole brain function. This approach would also help in better understanding PPS alterations in different psychiatric and neurodevelopmental conditions, as reviewed above.

The idea that the PPS system is inherently spatiotemporal is associated with novel empirical predictions, which may be tested in present and future computational works. On the behavioural side, this would imply that both spatial and temporal features of stimuli should affect PPS responses (e.g., reaction times). To some extent, this has been already shown through velocity or timing dependent modulations of PPS [20,40], but we expect this link to have implications at deeper, more fundamental, levels. For example, we expect a PPS response to be observed not only when a tactile stimulus is coupled with a simultaneous near external stimulus, but also at larger spatial distances when the delay between the two stimuli is compatible with ecological statistical regularities. Far approaching stimuli are usually coupled with touch after a given temporal delay (determined by the stimulus speed, acceleration, and higher order dynamical properties). Thus, we expect a maximal PPS response when touch is provided at the “most likely” delay following a certain type of external stimulus. On the neurophysiological side, we expect neurons with spatiotemporal RFs to be present in the PPS system. This would imply the existence of neurons tuned to specific temporal delays between touch and external stimuli (e.g., contact in 0, 100 or 200 ms) *and* to specific distances between the touched body part and the external stimulus. More generally, events with spatial or temporal features linked to potential contact should activate the PPS system and yield similar neural representations (measurable via EEG or fMRI) in PPS areas. These properties may already be tested in present computational works. In this sense, Bufacchi’s reinforcement learning model [64] arguably is the one which accounts more directly for temporal features, since *future* reward prediction is the key variable being optimised in their computational framework. Other models focusing on “pure” statistical regularity learning (e.g., [30]) are in their current form dealing only with spatial patterns, but can be adapted to embed temporal regularities as well by adding recurrent connections (as in [81]). In general, dealing with temporal features in ANNs (and mathematical models) is notoriously more difficult than with spatial features [82]. However, the additional predictive power resulting from a paradigm shift toward spatiotemporal modelling is likely worth the effort.

## 4. Conclusions

PPS is a rich and multifaceted neuroscientific concept which has driven numerous experimental and theoretical works since the late 1970s. Here, through the lens of David Marr’s three levels of description, we reviewed recent computational models which simulated PPS and its key properties. We believe that reasoning at different description levels, from the most basic neural implementation to the abstract purpose of cognitive systems, critically helps in understanding the nervous system, and thus PPS encoding. We conclude by attempting to bridge models under an overarching functional definition of PPS as a neural interface processing body-environment relationships in space-time, thus allowing for predictions (short-term) and putatively planning (longer-term).

All living beings experience the world from a specific place in space and moment in time. Space and time, and whether they are independent or not, have been a matter of debate among philosophers since ancient times [83]. Modern physics entrenched the idea that space and time are intimately intertwined in a single continuum, leading to the concept of spacetime. Similarly, we suggest that space and time should be more often considered together for the proper interpretation of brain functions, given the fact that our brains are constantly exposed to spatial and temporal regularities together [84]. This is particularly evident when considering the functions enabled by the PPS system, mediating body-environment interactions. To properly convey this notion, we propose that PPS should actually be renamed Peripersonal SpaceTime - PPST. Future empirical work will provide evidence supporting (or challenging) this view.
